# Self-Reported Familiarity with and Consumption of Fermented Foods and Awareness of Probiotics: An Online Convenience Sample of Adults in Saudi Arabia

**DOI:** 10.3390/foods15213886

**Published:** 2026-10-30

**Authors:** Hathami Alsuhebani, Hend F. Alharbi

**Affiliations:** 1 Department of Food Science and Human Nutrition, College of Agriculture and Food, Qassim University, Buraydah 51452, Saudi Arabia; 2 Department of Clinical Nutrition, Medical City, Qassim University, Buraydah 52571, Saudi Arabia

**Keywords:** fermented foods, probiotics, consumer awareness, nutrition labels, Saudi Arabia, consumer behavior, cross-sectional survey

## Abstract

This descriptive cross-sectional study surveyed an online convenience sample of 689 adults from five regions of Saudi Arabia regarding their familiarity with and consumption of 13 fermented foods, probiotic awareness, perceived health benefits, purchasing, and nutrition-label use. Chi-square tests were used to examine associations with educational level, and binary logistic regression was used to identify sociodemographic factors independently associated with familiarity. The sample was disproportionately female (62.3%) and highly educated (64.2% bachelor’s, 27.1% diploma or less, and 8.7% postgraduate). Only 23.2% of participants reported knowing what probiotics were, 52.4% reported familiarity with fermented foods, and 86.2% did not know whether all fermented foods contained probiotics. Laban and its varieties, yogurt, Greek yogurt, and commercial probiotic dairy products were the most familiar and consumed foods, whereas tempeh, miso, and kefir were the least familiar. Female gender (adjusted odds ratio [OR] 2.44, 95% confidence interval [CI] 1.76–3.39) and higher educational attainment (bachelor’s: OR 1.60, 95% CI 1.11–2.30; postgraduate: OR 2.20, 95% CI 1.18–4.09) were independently associated with self-reported familiarity with fermented foods. These findings suggest limited self-reported awareness of probiotics in this sample. Consumer education and clearer information on live cultures may be worth exploring, but their effectiveness needs evaluation. Due to the convenience sampling and self-reported measures, these findings should not be generalized to the entire Saudi adult population.

## 1. Introduction

Foods are increasingly recognized not only as sources of energy and essential nutrients but also as components of dietary patterns that may influence health and the risk of chronic diseases [1]. This broader perspective has contributed to the growing interest in functional foods and foods that provide health-related benefits beyond basic nutrition [2]. Among these foods, fermented products are diverse and historically important. Fermentation involves the intentional growth and metabolic activity of microorganisms and the enzymatic transformation of food components, resulting in changes in sensory characteristics, preservation, and nutritional properties [3]. Fermented foods, including dairy, vegetables, cereals, legumes, meat, and beverages, are consumed across cultures. Their diversity reflects substantial variations in raw materials, microorganisms, processing conditions, and traditional practices [4].

Fermentation can influence food characteristics by producing or transforming organic acids, peptides, vitamins, volatile compounds, and other metabolites. It may also alter the digestibility, bioavailability, and sensory properties of food components [3,4]. However, the health implications of fermented foods are not uniform and depend on the food matrix, microorganisms involved, processing conditions, and the extent to which viable microorganisms remain at the time of consumption [5,6].

Probiotics are live microorganisms that confer health benefits to the host when administered in adequate amounts [7]. Therefore, the term fermented food should not be considered synonymous with probiotic food. Although some fermented foods may contain microorganisms with probiotic potential, fermentation alone does not establish whether a food is a probiotic product. Some fermented foods are heated or pasteurized after fermentation. In contrast, others may contain microorganisms that have not been sufficiently characterized or demonstrated to provide health benefits under the required conditions [3,5].

The classification of foods, such as pickles, as probiotic foods is debated. Some pickles are produced by lactic acid fermentation and may contain live microorganisms, whereas others are acidified with vinegar or pasteurized and contain few or no viable cells [3,6]. Lactic acid-fermented vegetables harbor diverse lactic acid bacteria and have been proposed as potential sources of probiotics [8]. Others argue that even when live microorganisms are present, a food qualifies as a probiotic only if the specific strains have been characterized and shown to confer a health benefit, and that foods with undefined live microorganisms are better described as containing live cultures [3,7]. This distinction is important for consumer communication because the increasingly prominent health-related marketing of fermented and probiotic products may encourage consumers to interpret these two categories as equivalent.

Interest in fermented foods has increased internationally; however, consumer understanding of these foods appears to be variable. A recent US online survey of 751 consumers found that yogurt was the most frequently recognized fermented food. Respondents also associated a broad range of fermented foods with potential health and probiotic benefits [9]. These findings suggest that familiarity with fermented foods may be strongly influenced by culturally familiar products. Consumers may have difficulty distinguishing fermentation, live microorganisms, and probiotics.

Saudi Arabia provides an informative setting for investigating these issues in several ways. Fermented dairy products and other traditionally fermented foods have long been incorporated into the food culture of Saudi Arabia and the Arabian Peninsula [10]. Obesity and type 2 diabetes represent substantial public health challenges in the country [11,12], increasing interest in dietary approaches that may support healthier eating patterns. However, recommendations concerning fermented foods should be based on accurate consumer understanding rather than the assumption that all fermented foods have equivalent health effects. In previous Saudi studies, most adults associated probiotics and prebiotics with gut health [13], probiotic awareness was moderate, with yogurt the most recognized source and limited knowledge and cost the main reported barriers [14], and the mean nutrition-label knowledge score was 45% [15]. These studies did not assess familiarity with or consumption of a range of specific fermented foods or whether consumers distinguish fermented foods from probiotics.

The study aimed to assess how familiar adults in Saudi Arabia were with fermented foods and whether they consumed them, in an online convenience sample. The secondary descriptive aims covered participants’ awareness of probiotics, probiotic supplement use, and whether they distinguished fermented foods from probiotic foods. The study also assessed perceived health benefits, reasons for consumption or non-consumption, information sources, purchasing practices, nutrition-label use, and sociodemographic factors independently associated with self-reported familiarity.

## 2. Materials and Methods

### 2.1. Study Design and Participants

This descriptive cross-sectional study was conducted among adults residing in Saudi Arabia. A total of 689 participants were recruited from five geographic regions of the country: Central, Western, Eastern, Northern, and Southern. Participants were recruited using a convenience sampling approach through an electronically distributed self-administered questionnaire over a six-month period from April to September 2022. The questionnaire link was distributed through social media platforms, messaging applications, and institutional networks, and recipients were able to forward it to other participants. As the total number of individuals reached could not be determined, the response rate could not be calculated. Eligible participants were adults aged 18 years or older who resided in Saudi Arabia and could complete the questionnaire independently. All 689 participants who completed the questionnaire met the eligibility criteria, and no responses were excluded from the final analysis. The sample size was calculated using Raosoft (Raosoft Inc., Seattle, WA, USA), assuming a 95% confidence level, 50% response distribution, and 5% margin of error, which indicated a minimum requirement of 385 participants in the study. Although the final group of 689 participants surpassed this requirement, this calculation assumes random sampling and does not make the convenience sample representative. Participation was voluntary and anonymous. Electronic informed consent was obtained before accessing the questionnaire. The study protocol was reviewed and approved by the Committee of Research Ethics, Deanship of Scientific Research, Qassim University (approval no. 21-14-16) on 28 March 2022. All procedures were conducted in accordance with the principles of the Declaration of Helsinki [16].

### 2.2. Questionnaire Development

**Figure 1:**
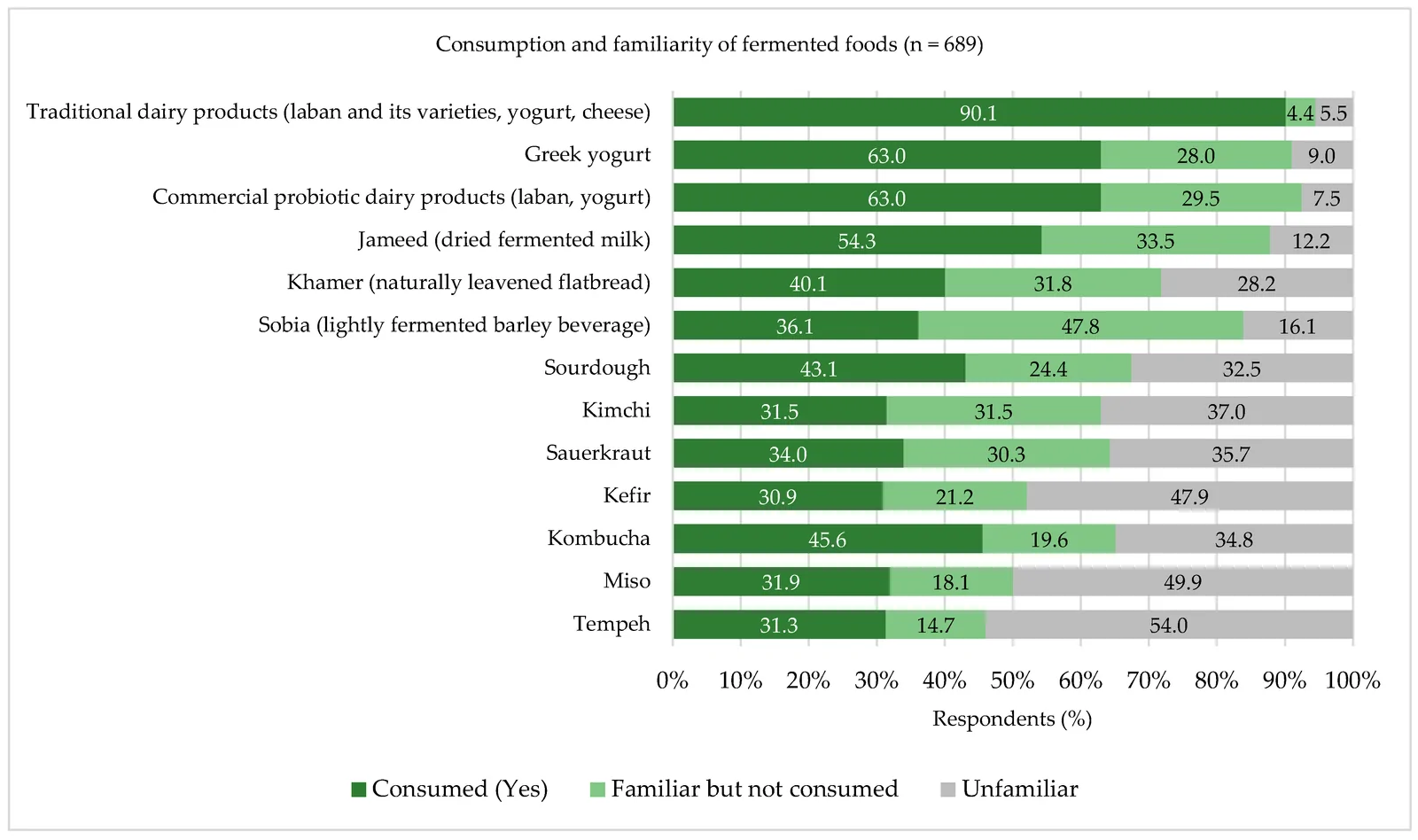
Self-reported familiarity with and consumption of 13 fermented foods. Bars show the percentage of participants (*n* = 689) selecting each of the three mutually exclusive response categories for every food item: consumed, familiar but not consumed, and unfamiliar. Percentages may not total 100% because of rounding.

Data were collected using a structured, self-administered questionnaire (S2) drafted directly in Arabic and delivered via Google Forms (Google LLC, Mountain View, CA, USA) and comprising three sections. The first section collected sociodemographic information, including age, gender, marital status, place of residence, region, educational level, monthly income, and occupation. The second section assessed the participants’ familiarity with fermented foods and awareness of probiotics, perceived health benefits, and sources of information about fermented foods and probiotics. The third section assessed consumption and consumer behavior. Participants were asked about their familiarity with and consumption of 13 selected fermented food products: traditional dairy products (laban and its varieties [traditional fermented milk], yogurt, and cheese), Greek yogurt, commercial probiotic dairy products (laban and yogurt), jameed (dried fermented milk), khamer (naturally leavened flatbread), sobia (lightly fermented barley beverage), sourdough, kimchi, sauerkraut, kefir, kombucha, miso, and tempeh (Figure 1). The 13 items selected included both traditional Saudi fermented foods and international fermented products available or consumed in Saudi Arabia and were presented to all participants in the order shown in Figure 1. Brief descriptions of selected items were included to aid product identification; no general definition of fermented foods was provided after the familiarity question. Participants were also asked about the reasons for consuming fermented foods and, using the item “What is your reason for not consuming these foods?”, for not consuming them; this item did not specify which foods participants were referring to. The questionnaire also covered probiotic supplement use, place of purchase or home preparation, presence of nutrition labels on purchased products, label reading and understanding, and influence of labels on purchasing. All items were mandatory; therefore, all 689 participants answered all items. The questions on reasons for consumption, reasons for non-consumption, and place of purchase allowed multiple responses, but each participant selected only one option per question; each was therefore coded as a single categorical variable.

Responses were recorded using nominal or three-point ordinal response categories, including “yes,” “no,” “sometimes,” and “do not know.” The questionnaire was developed by adapting and modifying items from prior studies. Items assessing familiarity with fermented foods were adapted from Aktaç et al. [17], questions on nutrition-label use and purchasing behavior from Alfawaz et al. [18], and items on perceived health benefits from Šikić-Pogačar et al. [19]. The perceived health benefit statements reflected health effects proposed for fermented foods, for which the strength of evidence varies. A fermented food diet increased gut microbiota diversity and reduced inflammatory markers in a randomized trial [20], whereas evidence for effects on body weight and mental health is limited and largely observational [21,22]. The statement that all fermented foods contain probiotics is incorrect and was included to test whether the participants distinguished fermented foods from probiotics [3,7]. These statements assessed perceptions and were not presented as established effects. The items were modified to fit the study objectives and the Saudi context, and were reviewed by specialists in food science and clinical nutrition to establish face and content validity. A formal pilot study was not conducted before data collection.

### 2.3. Statistical Analysis

Data were analyzed using SPSS (version 26.0; IBM, Armonk, NY, USA) and Excel (version 2205). Data were screened for completeness before statistical analysis. All variables, including age, were analyzed as categorical variables and are presented as frequency and percentage. Because the chi-square tests and logistic regression do not require normally distributed variables, normality testing was not applicable. Age was collected using four predefined response categories (18–29, 30–39, 40–49, and ≥50 years) to allow comparison across adult age groups; these categories were not based on clinical cut-offs. Associations between educational level and categorical responses were assessed using Pearson’s chi-square tests. When more than 20% of cells had expected counts below 5 or any cell had an expected count below 1, the Fisher–Freeman–Halton exact test with Monte Carlo estimation (10,000 sampled tables) was used to analyze the data. To account for multiple comparisons, *p*-values from the 19 tests reported in Tables 2 and 3 were adjusted using the Benjamini–Hochberg false discovery rate (FDR) procedure [23], calculated from unrounded Pearson *p*-values or Monte Carlo estimates for exact tests. Significance was set at an FDR-adjusted *p* < 0.05, and the more conservative Holm correction was applied as a sensitivity analysis [24].

Binary logistic regression was performed to identify factors independently associated with self-reported familiarity with fermented foods. Age group, gender, and educational level were entered as covariates a priori based on previously reported sociodemographic differences in probiotic awareness and attitudes [13,25]; other sociodemographic variables were not included to limit model complexity. No stepwise variable selection was used. The results are presented as odds ratios (ORs) with 95% confidence intervals (CIs). The significance for the regression model was set at a two-sided *p* < 0.05.

Multiple correspondence analysis (MCA) was used to explore patterns of familiarity and consumption across the 13 fermented food items (active variables), each coded as consumed, familiar but not consumed, or unfamiliar. A two-dimensional solution was obtained using variable principal normalization (SPSS Optimal Scaling procedure). Age, gender, educational level, sources of information about probiotics and fermented foods, nutrition-label reading and understanding, and reasons for consumption were included as supplementary variables and did not contribute to the construction of the dimensions. Patterns were examined using a joint category plot and discrimination measures.

## 3. Results

### 3.1. Sociodemographic Characteristics

This study included 689 adults. Women represented 62.3% of the sample (*n* = 429), while men represented 37.7% (*n* = 260). Participants were distributed across four age groups, with the largest proportions aged 30–39 years (27.4%) and ≥50 years (27.0%). Most participants were married (69.1%) and lived in urban areas (61.7%). The largest proportion of participants was recruited from the Central region (46.2%), followed by the Western (20.6%), Eastern (15.7%), Northern (10.6%), and Southern (7.0%) regions. Regarding education, 64.2% held a bachelor’s degree, 27.1% had a diploma or less, and 8.7% had postgraduate degrees. Almost half of the participants were employed (46.7%). The participants’ detailed sociodemographic characteristics are shown in Table 1. The sample was disproportionately female and highly educated and was not representative of the adult population of Saudi Arabia.

### 3.2. Familiarity with Fermented Foods and Awareness of Probiotics

Table 2 presents the participants’ self-reported familiarity with fermented foods, awareness of probiotics, probiotic supplement use, and perceived health benefits of fermented foods across different educational levels. These measures reflected participants’ reported familiarity, behaviors, and perceptions, rather than objectively assessed nutritional knowledge. Only 160 participants (23.2%) reported knowing what probiotics were. Self-reported probiotic awareness increased with educational attainment, from 15.0% among participants with a diploma or less to 24.0% among bachelor’s graduates and 43.3% among postgraduates (*p* < 0.001). In comparison, 361 participants (52.4%) reported knowing what fermented foods were. Self-reported familiarity increased from 42.8% among participants with a diploma or less to 55.0% among bachelor’s degree graduates and 63.3% among postgraduates (*p* = 0.004). The use of probiotic supplements was uncommon in this study. Thirteen participants (1.9%) reported current use, and 66 (9.6%) reported occasional use. Supplement use did not differ significantly according to educational level (*p* = 0.222). A substantial proportion of the participants were unable to determine whether all fermented foods contained probiotics. Overall, 594 participants (86.2%) selected “do not know,” 67 (9.7%) correctly indicated that not all fermented foods contain probiotics, and 28 (4.1%) incorrectly indicated that all fermented foods contain probiotics (*p* < 0.001).

Familiarity with and consumption of the 13 fermented foods varied widely. Fermented dairy products—laban and its varieties, yogurt, Greek yogurt, and commercial probiotic dairy products—were the most widely known and consumed, whereas tempeh, miso, and kefir were the least familiar products (Figure 1).

**Figure 2:**
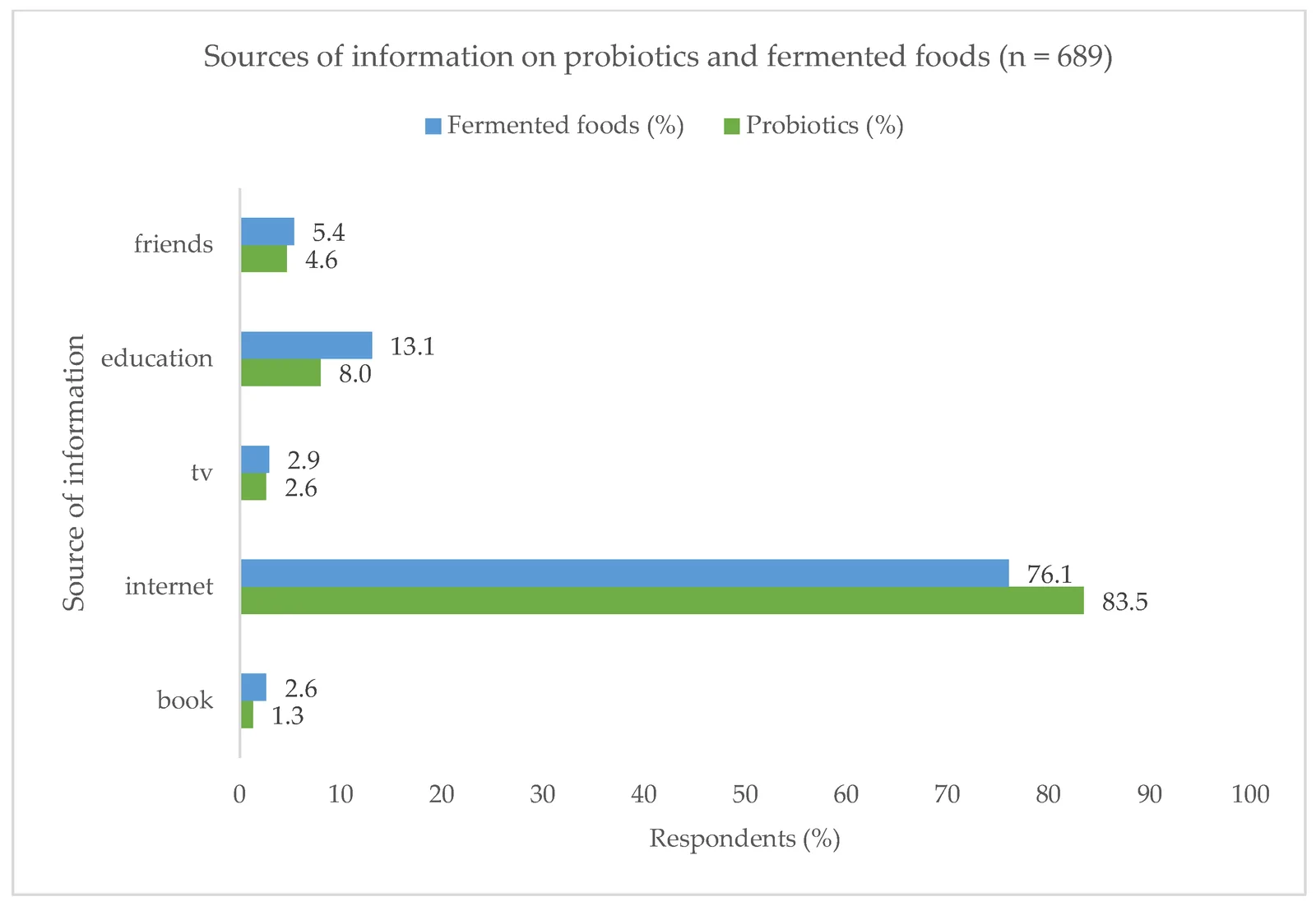
Principal source of information about probiotics and fermented foods (*n* = 689). Percentages may not total 100% because of rounding.

For perceived health benefits, “do not know” was the most frequent response across all assessed outcomes. The most frequently reported perceived benefit was helping to balance beneficial bacteria in the digestive system (43.8%), followed by reducing symptoms of digestive disorders (35.0%), supporting immunity and reducing inflammation (28.9%), and other perceived benefits (Table 2). The reporting of several perceived benefits differed significantly by educational level. However, differences were not statistically significant for perceived weight-loss effects (*p* = 0.095) or protection against environmental or fungal poisoning (*p* = 0.155). The Internet was the most frequently reported source of information about fermented foods and probiotics, followed by formal education and family or friends (Figure 2).

### 3.3. Consumption and Consumer Behavior

The most frequently reported reason for consuming fermented foods was perceived health benefits (32.1%), followed by cultural tradition (27.7%), taste (25.8%), and long shelf life (14.4%). The distribution of consumption motivations differed significantly according to educational level (*p* = 0.012; Table 3). When asked about reasons for non-consumption, 55.6% of participants selected “Do not know the benefit,” followed by price (21.8%) and lack of availability (21.2%). However, because all participants answered this question, including those who reported consuming fermented foods, these responses should not be interpreted as reasons for complete avoidance of fermented foods. Dislike of taste was reported by only 1.5% of participants. Lack of awareness of health benefits was reported by 65.8% of participants with a diploma or less compared with 46.7% of postgraduates (*p* = 0.009). Fermented foods were primarily obtained from local shops (82.6%), while 11.0% reported preparing them at home and 6.4% obtained them from family markets. The place of purchase did not differ significantly by educational level (*p* = 0.311).

Nutrition label use was variable. Approximately one-third of the participants (34.1%) reported that the fermented products they purchased carried nutrition labels, while 35.3% reported that labels were present only sometimes, and 30.6% reported that labels were absent. Regular label reading was reported by 29.5%, occasional reading by 44.8%, and no label reading by 25.7% of participants. The distribution differed significantly by educational level (*p* = 0.001). Understanding of the information presented on nutrition labels was reported by 31.9%, and 42.1% reported understanding labels sometimes. Self-reported understanding differed by educational level (χ^2^(4) = 29.479, *p* < 0.001): 33.2% of participants with a diploma or less, 27.4% of bachelor’s graduates, and 61.7% of postgraduates reported understanding labels, so understanding was highest among postgraduates but did not increase steadily with education (Table 3). All 13 associations with *p* < 0.05 in Table 2 and Table 3 remained significant after FDR correction (adjusted *p* ≤ 0.017). Under the Holm correction, the associations with the perceived mental-health benefit and with reasons for consumption and non-consumption were not significant.

Nutrition labels influenced the purchasing decisions of 38.9% of the participants and sometimes influenced the decisions of an additional 40.8%; this distribution did not differ significantly by educational level (*p* = 0.080).

MCA was conducted on 13 fermented food variables, each with three response categories. Dimension 1 (inertia = 0.624) accounted for 31.19% of the total inertia and Dimension 2 (inertia = 0.421) for 21.05%, together 52.24%. The joint category plot (Figure S1) is presented as an exploratory description of familiarity and consumption patterns. In the joint category plot, consumed categories were generally positioned on the positive side of Dimension 1, whereas the familiar-but-not-consumed and unfamiliar categories were located in the upper-left and lower-left regions, respectively (Figure S1). Kefir (0.839), kimchi (0.834), tempeh (0.820), miso (0.813), and sauerkraut (0.798) had the highest discrimination measures on Dimension 1. Discrimination measures for the supplementary variables were low on both dimensions; the highest was for education on Dimension 1 (0.080), so the MCA provided little evidence that these characteristics distinguished consumption profiles.

### 3.4. Factors Associated with Self-Reported Familiarity

In the adjusted logistic regression model, gender and educational attainment were independently associated with self-reported familiarity with fermented foods, whereas age group was not (Table 4). Women had higher odds of reporting familiarity with fermented foods than men (adjusted OR 2.44, 95% CI 1.76–3.39; *p* < 0.001). Compared with participants with a diploma or less, those with a bachelor’s degree had higher odds of reporting familiarity (OR 1.60, 95% CI 1.11–2.30; *p* = 0.011), while those with postgraduate qualifications had higher odds (OR 2.20, 95% CI 1.18–4.09; *p* = 0.013). Age group was not significantly associated with familiarity (overall *p* = 0.239). None of the age groups differed significantly from the 18–29-year reference group.

## 4. Discussion

This study examined familiarity with and consumption of fermented foods and awareness of probiotics in an online convenience sample of adults in Saudi Arabia. In this sample, self-reported familiarity with fermented foods exceeded self-reported awareness of probiotics, and most participants could not determine whether all fermented foods contained probiotics. Consumption was concentrated in familiar dairy products, and lack of awareness of health benefits was the most frequently reported reason for non-consumption, although all participants answered this item. The Internet was the main source of information. Female gender and higher education were associated with self-reported familiarity.

### 4.1. Sociodemographic Differences in Fermented Food Familiarity and Probiotic Awareness

The difference between self-reported familiarity with fermented foods and awareness of probiotics highlights an important measurement issue. This study used a single self-report question to assess whether participants knew what fermented foods were. The 52.4% estimate should be interpreted as self-reported familiarity, rather than objectively demonstrated knowledge [9]. The logistic regression model identified characteristics associated with participants’ perception that they knew fermented foods; it did not identify predictors of objectively measured fermented food knowledge.

Previous research has suggested that consumers may have difficulty identifying fermentation and distinguishing fermented foods from other cultured or processed products [9]. Research in Slovenia has also identified substantial variation in public understanding of fermentation and its microorganisms [19]. Future studies should develop a multidimensional fermented food knowledge scale that incorporates fermentation processes, microbial viability, and evidence-based health effects.

Female gender and higher educational attainment were independently associated with self-reported familiarity with fermented foods, whereas age was not. The association with educational attainment is consistent with a Saudi community survey in which the education level was associated with food-related knowledge scores [26].

The higher odds observed among women should be interpreted cautiously. Previous research in Saudi adults has also reported gender differences in probiotic attitudes and preferences [25] and in food-related knowledge [26], and higher probiotic knowledge among more educated adults [14]. However, the mechanisms underlying the gender association remain uncertain. Differences in food purchasing, household food preparation, exposure to nutrition information, or health-information seeking may contribute, but these explanations were not directly assessed in the present study. The cross-sectional design does not permit causal interpretation of the gender or education associations.

### 4.2. Consumption of Fermented Foods

Consumption and recognition were concentrated among familiar dairy products, whereas tempeh, miso, and kefir were among the least familiar items. This pattern is consistent with Saudi and international research demonstrating the central role of yogurt and other dairy products in consumer recognition of fermented foods [14,17]. In a recent US survey by Hanlon et al., yogurt was recognized by 87.6% of the participants, substantially exceeding the recognition of other fermented food categories [9]. The present findings may reflect the established cultural position of fermented dairy products in the local food environment [10], although their commercial availability was not evaluated.

Perceived health benefits and cultural traditions were the two most frequently reported motivations for consumption, followed by taste and shelf life, in contrast to the US survey, in which taste was the leading motivation [9]. Lack of awareness of the health benefits was the most frequently cited reason for non-consumption (55.6%), although all participants, including consumers, answered this item. This response may reflect either a genuine knowledge gap or low perceived relevance of fermented foods; however, the questionnaire did not distinguish between the two. The cross-sectional design cannot establish whether increasing awareness raises consumption.

### 4.3. Sources of Information

The Internet was the most frequently reported source of information about fermented foods and probiotics, followed by formal education and family or friends. Digital channels are therefore a plausible route for evidence-based consumer education [26,27], provided that the content distinguishes fermented foods from probiotics and its effectiveness is evaluated.

### 4.4. Nutrition-Label Use and Understanding

Including “sometimes” responses, 74.3% of participants reported reading nutrition labels and 74.0% reported understanding them; for “yes” responses alone, the figures were 29.5% and 31.9%. Self-reported label reading and understanding were therefore similar. Because both measures were self-reported, they did not establish whether participants could interpret nutrition information correctly [28,29]; future studies should include an objective assessment of label comprehension. Self-reported label understanding did not follow a consistent educational gradient: it was highest among postgraduates but lower among bachelor’s graduates than among participants with a diploma or less, unlike earlier evidence that consumers with lower educational attainment have the greatest difficulty interpreting nutrition labels [28]. Educational attainment alone may therefore not explain differences in perceived understanding, and the overall chi-square test did not establish which groups differed from one another.

The finding that only 34.1% of participants reported that purchased fermented products carried nutrition labels should not be interpreted as evidence of regulatory non-compliance. This study relied on consumer self-reports and did not involve the direct examination of food packages. Product-level audits are required to evaluate compliance with labeling requirements. For probiotic products, labels should identify the live microorganisms, including strain designation and viable counts (colony-forming units), because health effects are strain-specific and depend on an adequate number of viable microorganisms [7]. Future studies should evaluate the availability and clarity of this information on commercial product labels in Saudi Arabia. Similarly, the statistically significant but non-monotonic association between education and regular label reading (Table 3) should be interpreted with caution and may reflect sampling characteristics or response behavior.

### 4.5. Distinguishing Fermented Foods from Probiotics

Only 9.7% of participants correctly indicated that not all fermented foods contain probiotics, whereas 86.2% selected “do not know.” The probiotic designation applies to specific live microorganisms for which a health benefit has been demonstrated under defined conditions [7]. Fermented foods, by contrast, represent a broad category defined by the microbial transformation of food substrates. Some fermented foods contain viable microorganisms at consumption, whereas others may undergo processing that reduces microbial viability [3,5].

The present findings are consistent with recent international evidence showing that consumers frequently associate fermented foods with probiotic benefits [9]. However, uncertainty, rather than an explicitly incorrect belief, was the dominant response in this sample. Consumer education may therefore need to explain the differences between fermentation, live cultures, and probiotics rather than promote fermented foods as “probiotic foods.” Clearer live-culture labeling may also help consumers distinguish products containing viable microorganisms from those processed after fermentation, although this should be evaluated prospectively.

### 4.6. Strengths and Limitations

This study had two strengths. It assessed multiple dimensions of consumer behavior across 13 fermented foods, including locally consumed products that are rarely covered in the existing literature. Recruitment spanned all five geographic regions of Saudi Arabia, providing broader geographic coverage than that of many previous regional studies.

Several limitations should be considered. First, and most importantly, the convenience sampling strategy and online data collection limit representativeness, exclude adults without Internet access, and may have favored participants in the authors’ and respondents’ networks and those interested in nutrition. Because the response rate could not be calculated, non-response bias could not be assessed. The sample was disproportionately female and highly educated, and almost half of the participants were recruited from the Central region. Because both gender and educational attainment were associated with familiarity, estimates of familiarity and consumption cannot be extrapolated to the adult population of Saudi Arabia and probably overestimate familiarity. Second, the cross-sectional design precludes inference about temporal order or causation in the associations of sociodemographic characteristics with familiarity and consumption. Third, most variables were self-reported and may be subject to recall and social desirability bias, and the 13 foods were presented in the same order to all participants; therefore, order effects cannot be excluded. The primary outcome was a single self-reported familiarity question rather than a validated knowledge score. The questionnaire was reviewed for face and content validity but was not pilot-tested. Because the instrument comprised independent categorical items rather than multi-item scales, internal consistency was not applicable, and construct validity and test–retest reliability were not assessed. Fourth, three associations were not significant under the Holm correction (Section 3.3) and should be interpreted cautiously. Fifth, the questionnaire recorded only whether participants consumed each food, not consumption frequency, portion sizes, or quantities; therefore, the findings describe consumption status rather than habitual intake. Sixth, the item assessing reasons for non-consumption was answered by all participants, including those who consumed some fermented foods. Because the question did not identify specific products, it was not possible to determine whether responses referred to particular fermented foods or to fermented foods more generally.

## 5. Conclusions

In this sample, consumption was concentrated in familiar dairy products, whereas tempeh, miso, and kefir were the least familiar. Approximately half of the participants reported familiarity with fermented foods, whereas fewer than one-quarter reported knowing what probiotics were. Female gender (adjusted OR 2.44, 95% CI 1.76–3.39) and higher educational attainment (bachelor’s: adjusted OR 1.60, 95% CI 1.11–2.30; postgraduate: adjusted OR 2.20, 95% CI 1.18–4.09) were independently associated with self-reported familiarity, and lack of awareness of health benefits was the most frequently reported reason for non-consumption, although all participants, including consumers, answered this item.

These findings suggest a gap between familiarity with culturally familiar fermented foods and awareness of probiotics. Targeted nutrition education through digital channels and clearer live-culture labeling may be worth exploring, but their effect on knowledge or consumption requires prospective evaluation. Because of the online convenience sampling and reliance on self-reported measures, the findings cannot be generalized to the adult population of Saudi Arabia. Future studies should use nationally representative sampling, a validated knowledge instrument, and validated methods for assessing consumption frequency and quantity.

## Figures and Tables

**Table 1:** Sociodemographic characteristics of the study participants (*n* = 689).

Variables	Items	*n*	%
Age	18–29	163	23.7
	30–39	189	27.4
	40–49	151	21.9
	≥50	186	27.0
Gender	Male	260	37.7
	Female	429	62.3
Marital status	Single	154	22.4
	Married	476	69.1
	Divorced/widowed	59	8.6
Residence	Urban	425	61.7
	Rural (governorate/village)	264	38.3
Region	Central	318	46.2
	Western	142	20.6
	Eastern	108	15.7
	Northern	73	10.6
	Southern	48	7.0
Education	Diploma or less	187	27.1
	BSc	442	64.2
	Postgraduate	60	8.7
Income	6000 SR or less	257	37.3
	6000–11,000 SR	169	24.5
	11,000–16,000 SR	135	19.6
	16,000 SR or more	128	18.6
Occupation	Students	80	11.6
	Working	322	46.7
	Retired	104	15.1
	Not working	183	26.6

Values are presented as numbers (%). Percentages were calculated from the total sample size (*n* = 689). SR, Saudi Riyal; BSc, bachelor’s degree; Diploma or less, highest qualification at diploma level or below. Percentages may not total 100% because of rounding errors.

**Table 2:** Self-reported familiarity with fermented foods, awareness of probiotics, probiotic supplement use, and perceived health benefits of fermented foods by educational level (*n* = 689).

Variables	Total	Diploma or Less	BSc	Postgraduate	*p*-Value	FDR-Adjusted *p*
Know what probiotics are						
yes	160 (23.2)	28 (15.0)	106 (24.0)	26 (43.3)	<0.001	<0.001
no	529 (76.8)	159 (85.0)	336 (76.0)	34 (56.7)		
Consumes probiotic supplements						
yes	13 (1.9)	5 (2.7)	6 (1.4)	2 (3.3)	0.222	0.248
no	610 (88.5)	163 (87.2)	398 (90.0)	49 (81.7)		
Sometimes	66 (9.6)	19 (10.2)	38 (8.6)	9 (15.0)		
Know what fermented foods are						
yes	361 (52.4)	80 (42.8)	243 (55.0)	38 (63.3)	0.004	0.008
no	328 (47.6)	107 (57.2)	199 (45.0)	22 (36.7)		
All fermented foods contain probiotic bacteria.						
yes	28 (4.1)	3 (1.6)	17 (3.8)	8 (13.3)	<0.001	<0.001
no	67 (9.7)	27 (14.4)	35 (7.9)	5 (8.3)		
Do not know	594 (86.2)	157 (84.0)	390 (88.2)	47 (78.3)		
**Benefits of fermented foods**						
Helps balance beneficial bacteria in the digestive system						
yes	302 (43.8)	68 (36.4)	195 (44.1)	39 (65.0)	0.001	0.002
no	18 (2.6)	8 (4.3)	10 (2.3)	0 (0.0)		
Do not know	369 (53.6)	111 (59.4)	237 (53.6)	21 (35.0)		
Help reduce the symptoms of digestive disorders						
yes	241 (35.0)	41 (21.9)	166 (37.6)	34 (56.7)	<0.001	<0.001
no	43 (6.2)	22 (11.8)	20 (4.5)	1 (1.7)		
Do not know	405 (58.8)	124 (66.3)	256 (57.9)	25 (41.7)		
May help with weight loss						
yes	154 (22.4)	40 (21.4)	92 (20.8)	22 (36.7)	0.095	0.120
no	81 (11.8)	22 (11.8)	54 (12.2)	5 (8.3)		
Do not know	454 (65.9)	125 (66.8)	296 (67.0)	33 (55.0)		
Prevents/reduces the risk of chronic diseases						
yes	142 (20.6)	49 (26.2)	70 (15.8)	23 (38.3)	<0.001	<0.001
no	56 (8.1)	12 (6.4)	40 (9.0)	4 (6.7)		
Do not know	491 (71.3)	126 (67.4)	332 (75.1)	33 (55.0)		
Promote cardiovascular health						
yes	132 (19.2)	27 (14.4)	82 (18.6)	23 (38.3)	<0.001	<0.001
no	60 (8.7)	29 (15.5)	30 (6.8)	1 (1.7)		
Do not know	497 (72.1)	131 (70.1)	330 (74.7)	36 (60.0)		
Protects against environmental or fungal poisoning						
yes	155 (22.5)	47 (25.1)	89 (20.1)	19 (31.7)	0.155	0.185
no	42 (6.1)	14 (7.5)	24 (5.4)	4 (6.7)		
Do not know	492 (71.4)	126 (67.4)	329 (74.4)	37 (61.7)		
Boosts the body’s immunity and reduces inflammation						
yes	199 (28.9)	42 (22.5)	134 (30.3)	23 (38.3)	0.001	0.002
no	51 (7.4)	25 (13.4)	25 (5.7)	1 (1.7)		
Do not know	439 (63.7)	120 (64.2)	283 (64.0)	36 (60.0)		
Supports mental health						
yes	147 (21.3)	33 (17.6)	94 (21.3)	20 (33.3)	0.007	0.012
no	61 (8.9)	26 (13.9)	33 (7.5)	2 (3.3)		
Do not know	481 (69.8)	128 (68.4)	315 (71.3)	38 (63.3)		

Values are presented as *n* (column %); percentages were calculated within each educational group (diploma or less, *n* = 187; BSc, *n* = 442; postgraduate, *n* = 60) and within the total sample (*n* = 689). *p*-values were obtained using Pearson’s chi-square test, except for probiotic supplement use and the perceived benefit of balancing beneficial bacteria. Holm correction was additionally applied as a sensitivity analysis; its results are reported in the text. BSc, bachelor’s degree.

**Table 3:** Self-reported reasons for consumption and non-consumption, place of purchase, and nutrition-label use by educational level (*n* = 689).

Variables	Total	Diploma or Less	BSc	Postgraduate	*p*-Value	FDR-Adjusted *p*
Reason to consume						
taste	178 (25.8)	60 (32.1)	103 (23.3)	15 (25.0)	0.012	0.017
benefit	221 (32.1)	54 (28.9)	144 (32.6)	23 (38.3)		
traditional food	191 (27.7)	37 (19.8)	137 (31.0)	17 (28.3)		
long shelf life	99 (14.4)	36 (19.3)	58 (13.1)	5 (8.3)		
Reported reasons for non-consumption (all respondents)						
price	150 (21.8)	33 (17.6)	105 (23.8)	12 (20.0)	0.009	0.014
taste	10 (1.5)	0 (0.0)	8 (1.8)	2 (3.3)		
not available	146 (21.2)	31 (16.6)	97 (21.9)	18 (30.0)		
Do not know the benefit	383 (55.6)	123 (65.8)	232 (52.5)	28 (46.7)		
Where to buy						
local shop	569 (82.6)	161 (86.1)	362 (81.9)	46 (76.7)	0.311	0.328
family market	44 (6.4)	9 (4.8)	28 (6.3)	7 (11.7)		
made at home	76 (11.0)	17 (9.1)	52 (11.8)	7 (11.7)		
Are there nutritional labels on the products?						
yes	235 (34.1)	58 (31.0)	156 (35.3)	21 (35.0)	0.824	0.824
no	211 (30.6)	57 (30.5)	135 (30.5)	19 (31.7)		
sometimes	243 (35.3)	72 (38.5)	151 (34.2)	20 (33.3)		
Read the label						
yes	203 (29.5)	60 (32.1)	112 (25.3)	31 (51.7)	0.001	0.002
no	177 (25.7)	49 (26.2)	117 (26.5)	11 (18.3)		
sometimes	309 (44.8)	78 (41.7)	213 (48.2)	18 (30.0)		
Understand label						
yes	220 (31.9)	62 (33.2)	121 (27.4)	37 (61.7)	<0.001	<0.001
no	179 (26.0)	51 (27.3)	121 (27.4)	7 (11.7)		
sometimes	290 (42.1)	74 (39.6)	200 (45.2)	16 (26.7)		
Label influence to buy						
yes	268 (38.9)	79 (42.2)	157 (35.5)	32 (53.3)	0.080	0.109
no	140 (20.3)	37 (19.8)	94 (21.3)	9 (15.0)		
sometimes	281 (40.8)	71 (38.0)	191 (43.2)	19 (31.7)		

Values are presented as *n* (column %); percentages were calculated within each educational group (diploma or less, *n* = 187; BSc, *n* = 442; postgraduate, *n* = 60) and within the total sample (*n* = 689). Reasons for consumption, reasons for non-consumption, and place of purchase (S2, Questions 20–22) allowed multiple responses, but each participant selected only one option. *p*-values were obtained using Pearson’s chi-square test, except for reasons for non-consumption, for which the Fisher–Freeman–Halton exact test with Monte Carlo estimation (10,000 sampled tables) was used. False discovery rate (FDR)-adjusted *p*-values were calculated using the Benjamini–Hochberg procedure across the 19 comparisons in Table 2 and Table 3; significance was set at an FDR-adjusted *p* < 0.05. Holm correction was additionally applied as a sensitivity analysis; its results are reported in the text. BSc, bachelor’s degree.

**Table 4:** Binary logistic regression analysis of factors associated with self-reported familiarity with fermented foods (n = 689).

Variables	OR	95% CI	*p*-Value
**Age group**			0.239
18–29 years	Ref.	—	—
30–39 years	0.68	0.44–1.06	0.085
40–49 years	1.00	0.63–1.58	0.986
≥50 years	0.94	0.60–1.48	0.794
**Gender**			**<0.001**
Male	Ref.	—	—
Female	**2.44**	**1.76–3.39**	**<0.001**
**Educational level**			**0.011**
Diploma or less	Ref.	—	—
BSc	**1.60**	**1.11–2.30**	**0.011**
Postgraduate	**2.20**	**1.18–4.09**	**0.013**

OR, odds ratio; CI, confidence interval; Ref., reference category; BSc, bachelor’s degree. Outcome: self-reported familiarity with fermented foods (yes = 1, no = 0). ORs are mutually adjusted for age group, gender, and educational level. Bold values indicate *p* < 0.05.

## Data Availability

The raw data supporting the conclusions of this study will be made available by the authors upon reasonable request.

## Supplementary Materials

The following supporting information can be downloaded at: https://www.mdpi.com/article/10.3390/foods15213886/s1, Figure S1: Correspondence analysis of fermented food consumption status. Category points representing three response categories for each food (consumed, familiar but not consumed, and unfamiliar) are displayed along the first two dimensions using variable principal normalization. Dimension 1 accounted for 31.19% of the total inertia (inertia = 0.624), while Dimension 2 accounted for 21.05% (inertia = 0.421), for a combined 52.24%. The proximity of category points suggests similarities in response patterns and should be interpreted descriptively. S2: Study questionnaire.

## Abbreviations

The following abbreviations are used in this manuscript:

BSc	Bachelor’s degree
CI	Confidence interval
FDR	False discovery rate
MCA	Multiple correspondence analysis
OR	Odds ratio
SPSS	Statistical Package for the Social Sciences
SR	Saudi Riyal
