# Supplementary material for: Self-Reported Familiarity with and Consumption of Fermented Foods and Awareness of Probiotics: An Online Convenience Sample of Adults in Saudi Arabia
Source: Foods. 2026 Oct 30;15(21):3886. doi: 10.3390/foods15213886 (PMC13762717; doi:10.3390/foods15213886)
Supplement: Supplementary file 1 [file foods-15-03886-s001.zip › foods-4655733-supplementary/foods-4655733-supplementary.pdf]

Introduction and participant information

Peace be upon you. Dear participant: Thank you for participating in this questionnaire. The study aims to investigate the prevalence, trends, and predictors of fermented-food consumption and awareness of fermented foods among residents of the Kingdom of Saudi Arabia. Ethical approval number: 16-14-21. The questionnaire will take approximately five minutes. Accurate and honest answers are important for the study's success. Your participation contributes to scientific knowledge and serves your country. We confirm that all responses will remain strictly confidential and will be used for research purposes only.

Sociodemographic information

1. Age

- 18–29
- 30–39
- 40–49
- 50 years or older

2. Gender

- Male
- Female

3. Region

- Central region
- Western region
- Northern region
- Eastern region
- Southern region

4. Place of residence

- City
- Governorate / village

5. Educational level

- Diploma or less
- Bachelor's degree
- Postgraduate studies

6. Occupation

- Student
- Working
- Retired
- Not working

7. Income level

- SAR 6,000 or less
- SAR 6,000–11,000
- SAR 11,000–16,000
- SAR 16,000 or more

Restricted - مقيد

8. Marital status

- Single
- Married
- Divorced /widowed

Health information

9. Weight (kg) ....

10. Height (cm) ....

11. Do you have any chronic diseases?

- Yes
- No

Knowledge and consumption information

12. Do you know what probiotics are?

- Yes
- No

13. Do you take probiotic supplements?

- Yes
- No
- Sometimes

14. What are your sources of information about probiotics?

- Friend
- Education
- Television
- Internet
- Books

15. Do you know what fermented foods are?

- Yes
- No

16. What are your sources of information about fermented foods?

- Friend
- Education
- Television
- Internet
- Books

17. Do all fermented foods contain probiotics?

- Yes
- No
- Do not know

18. Benefits of fermented foods:

Restricted - مقيد

Help balance beneficial bacteria in the digestive system	Yes / No / Do not know
Help reduce symptoms of digestive disorders	Yes / No / Do not know
May help with weight loss	Yes / No / Do not know
Prevent or reduce the risk of chronic diseases	Yes / No / Do not know
Promote cardiovascular health	Yes / No / Do not know
Protect against environmental or fungal poisoning	Yes / No / Do not know
Boost the body's immunity and reduce inflammation	Yes / No / Do not know
Support mental health	Yes / No / Do not know

19. Do you consume these foods? *

Madhir or baql (aqit, jameed, jathi, kathi, and maris)	Yes and I consume / I know what it is but do not consume it / I do not know what it is
Sobia (a fermented barley drink popular in the Hijaz region)	Yes and I consume / I know what it is but do not consume it / I do not know what it is
Khamir (flatbread from southern Saudi Arabia)	Yes and I consume / I know what it is but do not consume it / I do not know what it is
Sourdough bread (naturally fermented bread)	Yes and I consume / I know what it is but do not consume it / I do not know what it is
Kimchi (Korean pickled cabbage)	Yes and I consume / I know what it is but do not consume it / I do not know what it is
Sauerkraut (pickled cabbage)	Yes and I consume / I know what it is but do not consume it / I do not know what it is
Traditional dairy products, such as laban, yogurt, and cheese	Yes and I consume / I know what it is but do not consume it / I do not know what it is
Greek yogurt	Yes and I consume / I know what it is but do not consume it / I do not know what it is
Activia products (laban and yogurt)	Yes and I consume / I know what it is but do not consume it / I do not know what it is
Kefir milk (also called Indian mushroom; a dairy product)	Yes and I consume / I know what it is but do not consume it / I do not know what it is
Kombucha (fermented green or black tea)	Yes and I consume / I know what it is but do not consume it / I do not know what it is
Miso (a type of Japanese seasoning used to prepare soup)	Yes and I consume / I know what it is but do not consume it / I do not know what it is
Tempeh (of Indonesian origin; a fermented soybean product)	Yes and I consume / I know what it is but do not consume it / I do not know what it is

20. What is your reason for consuming?

- Taste
- Benefits
- Traditional food
- Long shelf life

Restricted - مقيد

21. What is your reason for not consuming?

- Price
- Taste
- Not available
- I do not know their benefits

22. Where do you buy them?

- Local markets
- Family market
- I make them at home

23. Is there a nutrition label on the product?

- Yes
- Sometimes
- No

24. Do you read the information on the product's nutrition label?

- Yes
- Sometimes
- No

25. Do you understand the information on the product's nutrition label?

- Yes
- Sometimes
- No

26. Does the information on the nutrition label influence you when purchasing the product?

- Yes
- Sometimes
- No

Restricted - مقيد
